# A Novel Rat Model of Mild Pulmonary Hypertension Associated with Pulmonary Venous Congestion Induced by Left Pulmonary Vein Banding

**DOI:** 10.3390/ijms25052827

**Published:** 2024-02-29

**Authors:** Jonas Münks, Athiththan Yogeswaran, Tobiah Kevin Antoine, Leonhard Anton Blumrich, Peter Dorfmüller, Hossein Ardeschir Ghofrani, Birgit Assmus, Ralph Theo Schermuly, Akylbek Sydykov

**Affiliations:** 1 Excellence Cluster Cardio-Pulmonary Institute (CPI), Universities of Giessen and Marburg Lung Center (UGMLC), German Center for Lung Research (DZL), Justus Liebig University of Giessen, 35392 Giessen, Germany; jonas.muenks@innere.med.uni-giessen.de (J.M.); athiththan.yogeswaran@innere.med.uni-giessen.de (A.Y.); tobiah.k.antoine@med.uni-giessen.de (T.K.A.); leonhard.blumrich@innere.med.uni-giessen.de (L.A.B.); peter.dorfmuller@patho.med.uni-giessen.de (P.D.); ardeschir.ghofrani@innere.med.uni-giessen.de (H.A.G.); birgit.assmus@innere.med.uni-giessen.de (B.A.); ralph.schermuly@innere.med.uni-giessen.de (R.T.S.); 2 Department of Pathology, Universities of Giessen and Marburg Lung Center (UGMLC), Justus Liebig University of Giessen, 35392 Giessen, Germany; 3 Department of Cardiology and Angiology, Justus Liebig University of Giessen, 35392 Giessen, Germany

**Keywords:** pulmonary hypertension, pulmonary vein banding, animal model

## Abstract

Pulmonary hypertension (PH) associated with left heart disease (PH-LHD) is the most common form of PH. In PH-LHD, changes in the pulmonary vasculature are assumed to be mainly caused by pulmonary venous congestion. However, the underlying mechanisms of this form of PH are poorly understood. We aimed to establish a model of PH associated with pulmonary venous congestion. Wistar–Kyoto rats underwent partial occlusion of the left pulmonary vein to induce pulmonary venous congestion or sham surgery and were assessed at various time points post-surgery (3, 6, 9, 12 weeks). In vivo cardiopulmonary phenotyping was performed by using echocardiography along with heart catheterization. Histomorphometry methods were used to assess pulmonary vascular remodeling (e.g., wall thickness, degree of muscularization). Left pulmonary vein banding (PVB) resulted in mildly elevated right ventricular systolic pressure and moderate right ventricular hypertrophy. In PVB rats, small- and medium-sized pulmonary vessels in the left lung were characterized by increased wall thickness and muscularization. Taken together, our data demonstrate that left PVB-induced pulmonary venous congestion is associated with pulmonary vascular remodeling and mild PH.

## 1. Introduction

Pulmonary hypertension (PH) is a disorder characterized by pulmonary artery pressure elevation and is estimated to affect 1% of the global population [1,2]. Importantly, PH is very heterogeneous and is associated with a wide range of clinical conditions. Among them, left heart disease (LHD) represents the leading cause of PH. Furthermore, left-sided congestive heart failure (HF) is the most common type of HF. According to epidemiological data, PH is present in up to 80% of these patients [3,4]. Importantly, the presence of PH in LHD is associated with increased morbidity and mortality [5,6].

Although there have been considerable advances in therapeutics for pulmonary arterial hypertension, there are currently no approved therapeutic strategies available for PH associated with LHD (PH-LHD). Despite this unmet medical need in this large group of PH patients, the limited number of animal models of PH-LHD has hampered advancing the understanding of its pathophysiology and development of new therapies [7]. While no model perfectly recapitulates the heterogeneity of PH-LHD in humans, a number of models have recently been proposed that provide mechanistic insight into how comorbidities known to associate with LHD may contribute to pulmonary vascular remodeling [8,9]. However, given the potent prognostic impact of PH in LHD patients, we sought to establish a novel model of PH-LHD in which the pulmonary vasculature is the main site of changes due to pulmonary congestion. A rat model of LHD consisted of surgical narrowing of the left pulmonary vein that recapitulates the most important features of HF, such as pulmonary congestion. This study included the identification of an appropriate degree of the pulmonary vein stenosis and characterization of the longitudinal changes due to pulmonary congestion caused by the left pulmonary vein banding in Wistar–Kyoto rats by means of hemodynamic evaluation, heart function assessment, and histomorphometric analysis of pulmonary vessels. This model might facilitate the exploration of the molecular mechanisms underlying PH-LHD and development of novel therapeutic strategies.

## 2. Results

### 2.1. Identification of an Appropriate Degree of Pulmonary Vein Stenosis

In the pilot trial, we first sought to identify the degree of pulmonary vein occlusion that would lead to the development of PH without causing significant mortality. For this purpose, different degrees of occlusion were applied in three separate sets of animals. In the first set, we subjected the left pulmonary vein to complete ligation. In the second and third sets, we induced stenosis of the left pulmonary vein to a degree of 0.6 mm and 0.8 mm, respectively. These experiments were conducted for four days, and then the animals were sacrificed, and the lungs were examined histologically. In the complete occlusion and 0.6 mm stenosis groups, the rats displayed progressive deterioration, causing some of the experiments to be prematurely terminated. In contrast, rats with the degree of the stenosis of the left pulmonary vein of 0.8 mm did not exhibit significant mortality.

**Figure 1:**
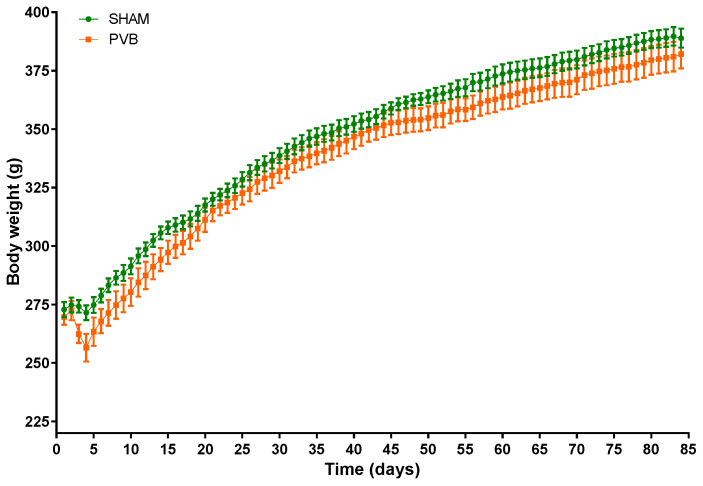
Body weight (BW) changes in rats subjected to left pulmonary vein banding (PVB) or sham surgery for 12 weeks. Values are means ± SEM, *n* = 9–10 rats per group.

After the identification of the required degree of stenosis of the left pulmonary vein diameter, we followed rats subjected to PVB for 12 weeks. During the first week, PVB rats showed greater weight reduction compared to sham animals (Figure 1). In the following weeks, however, PVB rats exhibited weight gain, which was not different from that in sham animals (Figure 1).

### 2.2. Time Course of Left Pulmonary Vein Banding

**Figure 2:**
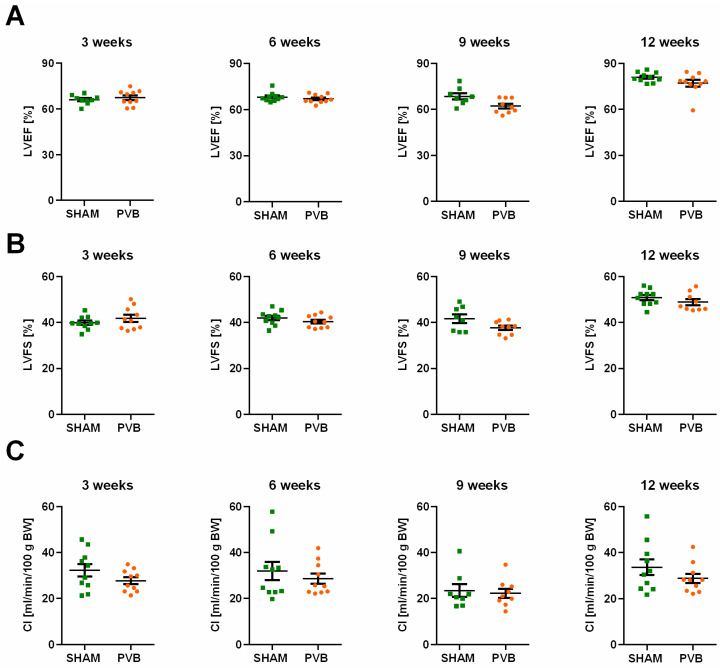
Echocardiographic characterization of the left ventricular function in rats subjected either to left pulmonary vein banding (PVB) or sham surgery for various time periods. (**A**) Left ventricular ejection fraction (LVEF). (**B**) Left ventricular fractional shortening (LVFS). (**C**) Cardiac index (CI). Values are means ± SEM. *n* = 7–10 rats per group.

Next, we aimed to investigate the time course of hemodynamic and histological pulmonary vessel changes at various time points following PVB: 3, 6, 9, and 12 weeks. It is noteworthy that left ventricular systolic function remained preserved in PVB rats at all timepoints, as evidenced by no changes in left ventricular ejection fraction (LVEF), left ventricular fractional shortening (LVFS), and cardiac index (CI) (Figure 2).

**Figure 3:**
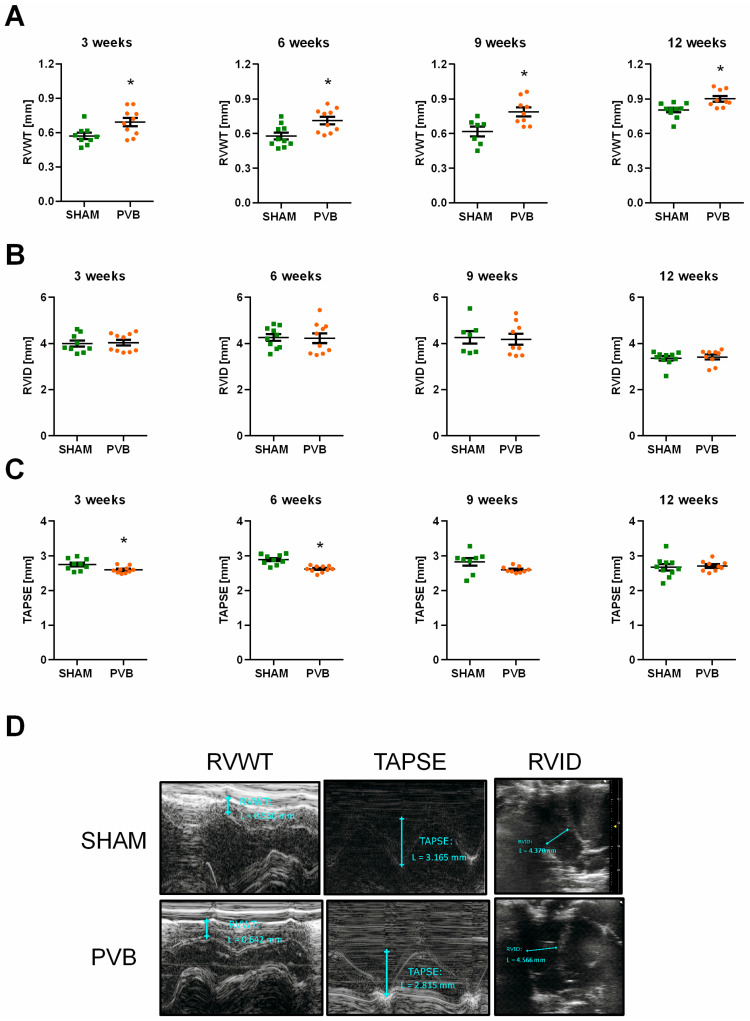
Echocardiographic characterization of the right ventricular structure and function in rats subjected to left pulmonary vein banding (PVB) or sham surgery for various amounts of time. (**A**) Right ventricular wall thickness (RVWT). (**B**) Right ventricular internal dimension (RVID). (**C**) Tricuspid annular plane systolic excursion (TAPSE). (**D**). Representative echocardiographic images from rats at 6 weeks after PVB/Sham surgery, which show right ventricular hypertrophy and mild right ventricular systolic dysfunction. Values are means ± SEM. * *p* < 0.05, *n* = 7–10 rats per group.

PVB rats displayed slight but statistically significant right ventricular (RV) hypertrophy, as evidenced by increased RV wall thickness (RVWT) at all time points (Figure 3A). RV chamber size, mirrored by the RV internal dimension (RVID), was not altered at any time point in PVB rats compared to sham animals (Figure 3B). However, a slight reduction in RV systolic function, as evidenced by decreased tricuspid annular plane systolic excursion (TAPSE), was observed in PVB animals at 3 and 6 weeks (Figure 3C).

**Figure 4:**
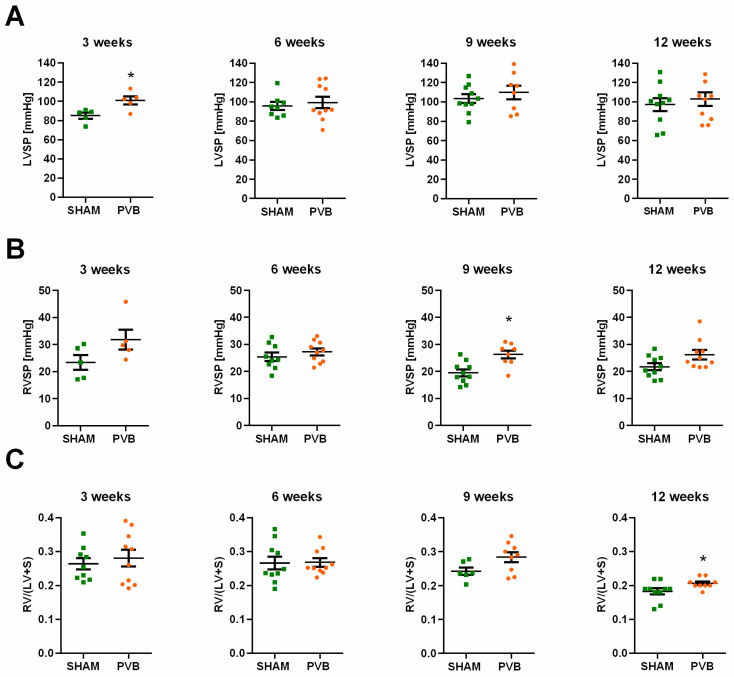
Characterization of hemodynamics and right ventricular hypertrophy in rats subjected to left pulmonary vein banding (PVB) or sham surgery for various times periods. (**A**) Left ventricular systolic pressure (LVSP). (**B**) Right ventricular systolic pressure (RVSP). (**C**) Right ventricle (RV) to left ventricle (LV) plus septum (S) weight ratio (RV/(LV + S)). Values are means ± SEM. * *p* < 0.05, *n* = 5–10 rats per group.

Hemodynamic measurements demonstrated no considerable differences in LV systolic pressure (LVSP) between PVB and sham rats (Figure 4A). Only after 3 weeks, LVSP values in PVB rats were slightly higher than those in sham rats (Figure 4A). RV systolic pressure (RVSP) values were slightly increased in PVB rats at 3 and 12 weeks (Figure 4B). After 9 weeks, RVSP values in PVB were significantly higher than those in sham animals (Figure 4B). The RV/(LV + septum) ratio values were slightly higher in PVB rats at 12 weeks compared to sham animals (Figure 4C). No changes in LV mass were observed in any group.

**Figure 5:**
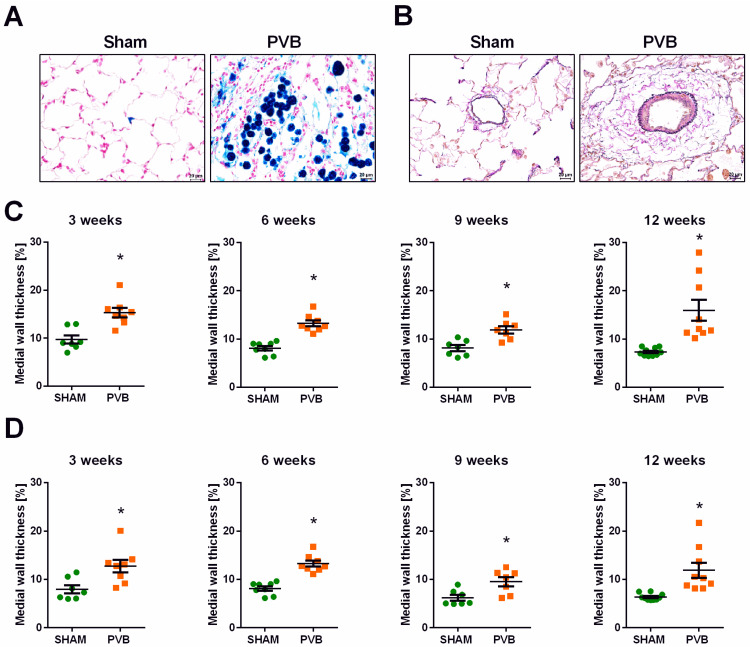
Pulmonary congestion in rats subjected to left pulmonary vein banding (PVB) and medial wall thickness in the left lungs of rats subjected to left PVB or sham surgery for various time periods. (**A**) Representative images of left lung sections showing the accumulation of heart failure cells or siderophages (greenish blue) in PVB lungs identified using a Prussian blue reaction. Images were taken at 400-fold magnification with a scale bar of 20 µm. (**B**) Representative images of left lung tissue sections from rats at 12 weeks after PVB/Sham surgery, which show thickened medial walls of pulmonary vessels in PVB rats. Quantification of the medial wall thickness was based on elastica-van-Gieson staining. Medial wall thickness was defined as the distance between the lamina elastica interna and the lamina elastica externa. Images were taken at 400-fold magnification with a scale bar of 20 µm. (**C**) Medial wall thickness of small pulmonary vessels (outer diameter 20–50 μm). (**D**) Medial wall thickness of medium-sized pulmonary vessels (outer diameter 50–100 μm). Values are means ± SEM. * *p* < 0.05, *n* = 7–10 rats per group.

Histological examinations revealed heart failure cells or siderophages in left lung sections of PVB rats (Figure 5A). To study remodeling of the pulmonary vessels in the left lungs of PVB rats, we performed histomorphometric examinations. First, we measured medial wall thickness (MWT) in small- (20–50 µm) and medium-sized pulmonary vessels (50–100 µm) in PVB and sham rats (Figure 5B–D). PVB rats exhibited a significant increase in MWT of small pulmonary vessels (20–50 µm) at all time points (Figure 5C). Similar findings were revealed in medium-sized pulmonary vessels (50–100 µm) (Figure 5D).

**Figure 6:**
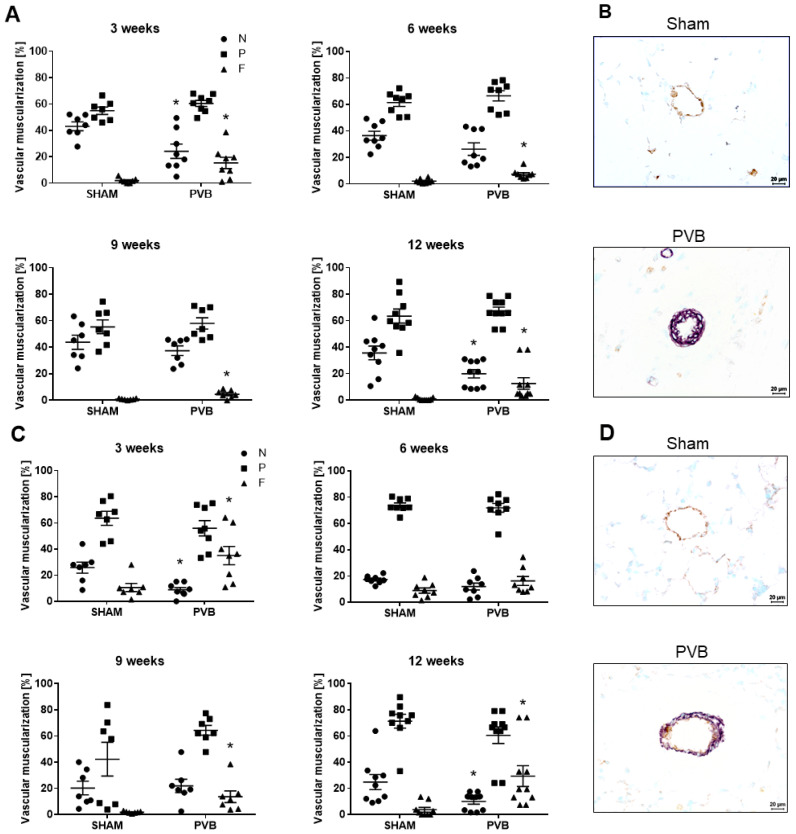
Muscularization of pulmonary vessels in left lungs of rats subjected to left pulmonary vein banding (PVB) or sham surgery for various time periods. The degree of vessel wall muscularization was determined for the quantification of pulmonary vascular remodeling. Vessels were categorized as non- (N), partially (P), or fully muscularized (F) after immunostaining against α–smooth muscle actin for detection of vascular smooth muscle cells (purple) and von Willebrand factor (vWF) for discrimination of endothelium (brown). (**A**) Degree of muscularization of small pulmonary vessels (outer diameter 20–50 μm). Values are means ± SEM. * *p* < 0.05, *n* = 7–10 rats per group. (**B**) Representative images of α-actin/vWF-stained left lung tissues from rats after 12 weeks of PVB/Sham surgery showing increased muscularization of small pulmonary vessels in PVB rats. Images were taken at 400-fold magnification with a scale bar of 20 µm. (**C**) Degree of muscularization of medium-sized pulmonary vessels (outer diameter 50–100 µm). Values are means ± SEM. * *p* < 0.05, *n* = 7–10 rats per group. (**D**) Representative images of α-actin/vWF stained left lung tissues from rats after 12 weeks of PVB/Sham surgery showing increased muscularization of medium-sized pulmonary vessels in PVB rats. Images were taken at 400-fold magnification with a scale bar of 20 µm.

Next, we assessed the degree of muscularization of small- (20–50 µm) and medium-sized (50–100 µm) pulmonary vessels (Figure 6A–D). The percentage of non-muscularized small and medium-sized vessels decreased at 3 and 12 weeks in PVB rats compared to sham animals (Figure 6A,C). In PVB rats, the percentage of fully muscularized vessels was increased at all time points, except the medium-sized vessels at 6 weeks (Figure 6A,C).

### 2.3. Involvement of Pulmonary Veins in Pulmonary Vessel Remodeling following Left Pulmonary Vein Banding

**Figure 7:**
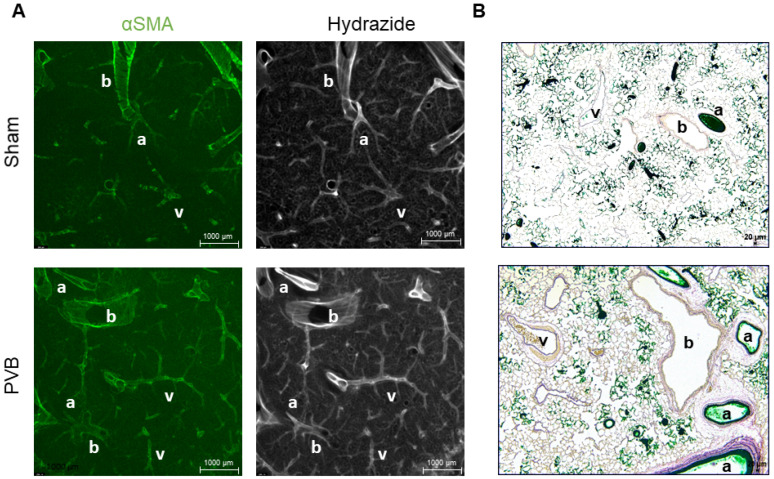
Increased muscularization of pulmonary veins in left lungs of rats subjected to left pulmonary vein banding (PVB) (**A**) Representative immunofluorescence images of lung sections (350 µm) of rats subjected to PVB or sham surgery for 9 weeks. Veins (V) of PVB lungs show increased staining for α-smooth muscle actin (αSMA) indicating increased muscularization compared to sham rats. Staining with hydrazide shows a greater abundance of elastic fibers in veins in PVB rats compared to sham animals. Scale bar 1000 µm. (**B**) Representative images of elastica-van-Gieson-stained lungs sections of rats subjected either to PVB or sham surgery for 9 weeks following injection of a green tissue marking dye through the pulmonary artery. All arteries up to the capillary bed contain ink, and veins remain intact. PVB rats display thickened walls in pulmonary veins. a = artery b = bronchus v = vein. Images were taken at 50-fold magnification with a scale bar of 20 µm.

Immunofluorescence microscopy of thick lung sections revealed increased muscularization and accumulation of elastic fibers in the pulmonary vessels of PVB rats compared to sham animals (Figure 7A). To differentiate between the arteries and veins, we injected a green tissue marking dye through the pulmonary artery. By this means, all arteries up to the capillary bed contain ink, while all the veins remain intact. From these sections, it was evident that the pulmonary veins in PVB rats were affected by the remodeling process, as indicated by their thickened walls (Figure 7B).

### 2.4. Contribution of Inflammatory Cells to the Remodeling of Pulmonary Vessels following Left Pulmonary Vein Banding

**Figure 8:**
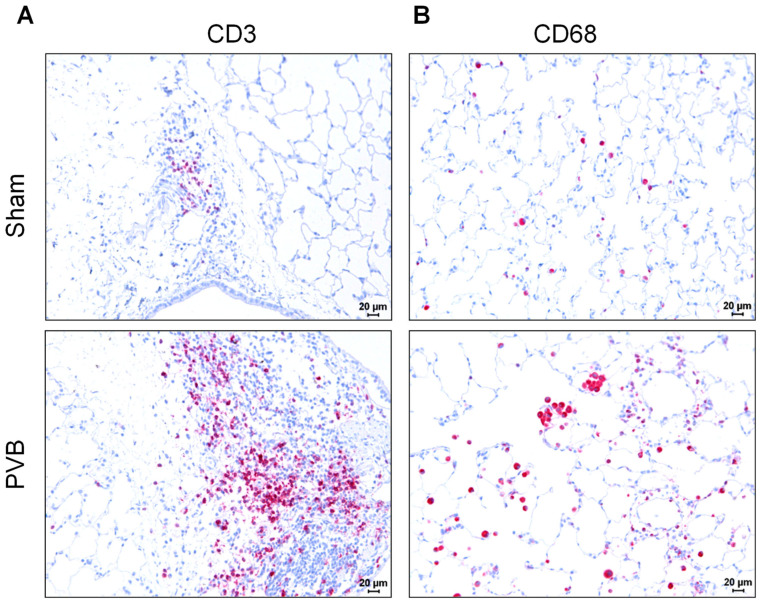
Increased accumulation of inflammatory cells in left lungs of rats subjected to left pulmonary vein banding (PVB) for 12 weeks. (**A**). Representative images showing staining with anti-CD3 antibodies. Images were taken at 200-fold magnification with a scale bar of 20 µm. CD3^+^ cells are stained pink (**B**) Representative images showing staining with anti-CD68 antibodies. Images were taken at 200-fold magnification with a scale bar of 20 µm. CD68^+^ cells are stained pink.

To gain insight into the potential involvement of inflammatory cells in the pulmonary vascular remodeling following PVB, we immunohistochemically examined the left lungs using antibodies against CD3 and CD68. Our analyses revealed a marked increase in infiltrating CD3-positive and CD68-positive cells in the lungs of PVB rats (Figure 8).

## 3. Discussion

In this study, we characterized longitudinal changes in cardiac function, hemodynamics, and pulmonary vascular morphology due to pulmonary congestion caused by left PVB in Wistar–Kyoto rats. We demonstrated that left PVB led to mildly elevated right ventricular systolic pressure and moderate right ventricular hypertrophy, as well as remodeling of small- and medium-sized pulmonary vessels in the left lung. The accumulation of inflammatory cells in the affected lungs suggests a possible contribution of inflammation to these changes.

PH-LHD is thought to occur as a result of increased back pressure due to left heart pathology [9]. In congestive HF, pulmonary congestion due to increased left atrial pressure results in blood accumulation and pressure elevation in pulmonary veins. Passive backpressure in the pulmonary circulation is followed by active vasoconstriction in pulmonary arteries due to the Hermo-Weiler or Kitaev reflex [10]. Persistent pressure elevation in pulmonary vessels may also be associated with subsequent pulmonary vascular remodeling. Currently available animal models of HF, in addition to left ventricular systolic or diastolic dysfunction, recapitulate various features of this condition determined by comorbidities, such as obesity, metabolic disorders, etc. [8,9]. However, as the main feature of congestive HF is pulmonary congestion and PH is the potent prognostic factor in these patients, we sought to establish a model of PH-LHD in which the changes in the pulmonary vasculature associated with pulmonary congestion are affected neither by left ventricular function nor by other comorbid factors. For this purpose, we established a rat model of left PVB.

In the experimental PVB model, the pulmonary veins are surgically constricted, causing obstruction of blood flow in the pulmonary circulation leading to elevated pulmonary venous pressure and an increase in pulmonary artery pressure (PAP). PVB was successfully applied in various large animals, such as calves [11], dogs [12], and piglets [13]. The porcine PVB is currently the most common large animal model for PH-LHD with a moderate elevation of PAP and pulmonary vascular remodeling [14,15,16,17,18]. However, PVB model in large animals has a number of disadvantages, including the requirement of multiple operators to perform the procedure, high maintenance costs, and longer duration of follow-up [8]. In contrast, the advantages of rat models are ease of maintenance, low operational and maintenance costs, and potential availability of various genetically modified animals.

The publications of PVB in rats are scarce. This might be due to technical challenges related to the small size of rats. Cottrill et al. [19] banded one of the pulmonary veins to reduce its external diameter to 0.8 mm. Eight weeks following the surgery, PVB rats developed moderate PH, as evidenced by mean PAP elevation [19]. Histological investigations of the vein-banded regions demonstrated venous congestion, arterialization of veins, and pulmonary arterial medial thickening [19]. These findings suggested that PVB in rats might be a suitable model for PH-LHD. However, due to the lack of studies, the exact order and timing of the functional, hemodynamic, and histologic events that underlie pulmonary vascular remodeling processes in this model remain unknown.

In the PVB model, pulmonary congestion is the major factor that initiates pulmonary vascular changes. Increased pressure in the pulmonary circulation may disrupt the integrity of the capillary endothelium allowing extravasation of erythrocytes. Subsequently, macrophages engulf and degrade extravasated erythrocytes, degrade heme proteins, accumulate iron, and become siderophages [20]. In the current study, pulmonary siderophages were detected in PVB rats with the Prussian blue reaction, which corroborated the presence of pulmonary congestion in these animals.

Importantly, pulmonary congestion in the PVB model develops due to stenosis of the pulmonary vein and is not associated with left heart pathology. This allows for studying the direct effects of pharmacological drugs on the pulmonary circulation in this model. Correspondingly, left ventricular function remained unaffected in PVB rats at all time points. PVB led to a mildly elevated right ventricular systolic pressure, which was lower than in a previous study [19]. However, it was sufficient to induce moderate right ventricular hypertrophy in PVB rats. The presence of PH in PVB rats was corroborated by findings of the remodeling of small and medium-sized pulmonary vessels in the left lung, which is evident as early as 3 weeks after stenosis induction. By using immunofluorescence microscopy of thick lung sections and the injection of a tissue-marking dye through the pulmonary artery, we demonstrated that at least some of the affected pulmonary vessels in PVB rats were pulmonary veins.

Animal models are valuable for investigating the effects of different drugs on the disease processes. According to our findings, therapeutic interventions in this model can be initiated as early as at three weeks post-PVB, when the pulmonary vascular remodeling and right ventricular hypertrophy have already developed. The duration of the potential treatment might depend on the specific research question and the mode of action of the particular drug. Nevertheless, any improvements in pulmonary vascular remodeling and right ventricular hypertrophy during the 12-week period post-PVB associated with the therapeutic interventions would indicate the positive effects of the treatment.

Elevated levels of circulating inflammatory cytokines were previously demonstrated in various animal models of pulmonary congestion in HF [9]. The accumulation of inflammatory cells in the affected lungs suggests a possible contribution of inflammation to the pulmonary vascular remodeling in PVB rats. This is in line with the findings of the previous study in the same model [19], which revealed sparse to moderate inflammation in vein-banded regions.

Extension of the observation period to several months will allow for the exploration of the evolution of pathological changes in this model. Further, experiments using rats with various genetic modifications or risk factors, such as aging, morbidity, and metabolic disorders, will allow not only the study of the pathogenetic mechanisms of pulmonary hypertension due to pulmonary congestion but also the exploration of the role of the confounding factors and the identification of new molecular therapeutic targets.

There are some limitations to the present study. Significant differences between various rat strains in susceptibility to pulmonary hypertension are widely recognized [21,22,23,24,25]. Therefore, our findings are applicable to Wistar–Kyoto rats only. Another important issue is the impact of various modifying factors. Pulmonary hypertension in patients with left heart disease is commonly associated with a variety of comorbidities, which might affect disease development and severity [7]. In our study, we induced pulmonary congestion in young healthy rats. Therefore, our findings cannot be directly translated into human situations. Nevertheless, our group is committed to characterizing this model in other rat strains and exploring the contribution of other potential risk factors like obesity, aging, metabolic disorders, genetic mutations, etc. in this model.

In summary, our work provided a detailed description of the longitudinal changes in pulmonary vascular morphology due to pulmonary congestion caused by left pulmonary vein banding in Wistar–Kyoto rats. Our data demonstrated that left PVB-induced pulmonary venous congestion causes pulmonary vascular remodeling and mild PH and, thus, might represent a suitable model to study PH-LHD. We believe that our model will contribute to a better understanding of the mechanisms that underlie PH-LHD and drive disease progression to develop novel, effective and safe pharmacological concepts.

## 4. Materials and Methods

Eight-week-old male Wistar–Kyoto rats (200–250 g) were purchased from Janvier Labs (Le Genest Saint Isle, France). The rats were kept in groups of 2–3 animals under appropriate barrier conditions in a 14/10 h light/dark cycle and received standard laboratory food (Altromin^®^, Altromin Spezialfutter GmbH & Co. KG, Lage, Germany) ad libitum and free access to water throughout the entire experiment. All experiments were approved by the governmental authorities (Regierungspräsidium Giessen, Giessen, Germany; Az. Gi 20/10, No. G5/2019) in accordance with German animal welfare law and European legislation for the protection of animals used for scientific purposes (2010/63/EU).

### 4.1. Experimental Design

To identify an appropriate grade of pulmonary vein stenosis, different degrees of occlusion were applied in three separate sets of animals. In the first set, the left pulmonary vein was subjected to complete ligation (*n* = 8). In the second and third sets, we induced stenosis of the left pulmonary vein to a degree of 0.6 mm or 0.8 mm (*n* = 8 for each group), respectively. These experiments were conducted for four days, and then the animals were sacrificed, and the lungs were examined histologically. All the subsequent PVB experiments were performed with a degree of stenosis of 0.8 mm.

After the identification of an appropriate grade of the pulmonary vein stenosis, rats were subjected to either PVB (*n* = 11) or sham surgery (*n* = 10) and were followed for 12 weeks. At the end of the observation period, rats were subjected to echocardiography and hemodynamic measurements. Afterward, the animals were sacrificed, and the lungs were examined histologically.

**Figure 9:**
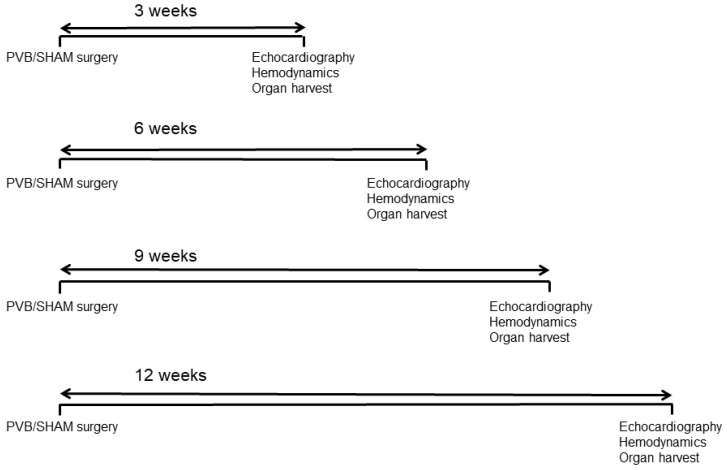
Schematic diagram of the experimental groups of rats subjected to left pulmonary vein banding or sham surgery and followed for various times.

To assess the time course of changes in the heart and pulmonary vasculature induced by PVB, rats were subjected to either PVB (*n* = 10 for each time point) or sham surgery (*n* = 10 for each time point) and were sacrificed 3, 6, 9, or 12 weeks after surgical procedures (Figure 9).

### 4.2. Model of Pulmonary Vein Banding

**Figure 10:**
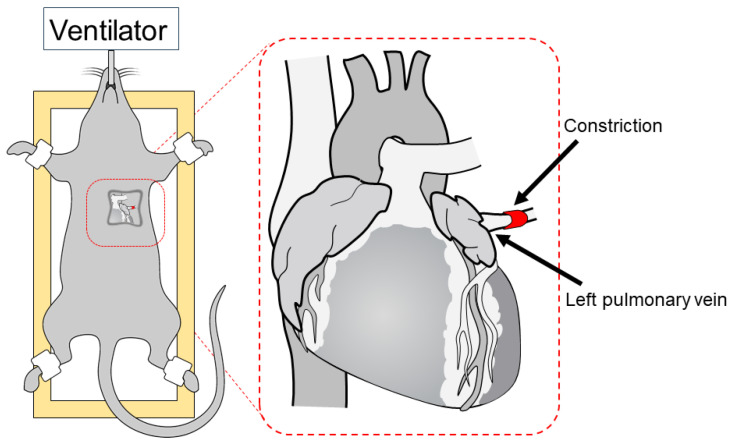
Schematic representation of the left pulmonary vein banding surgery.

Prior to surgery, buprenorphine hydrochloride (0.05 mg/kg bw, Buprenodale^®^, Albrecht GmbH, Aulendorf, Germany) was administered s.c. as an analgesic. Surgery was performed under general anesthesia using isoflurane (3% *v*/*v*, Isofluran Baxter^®^, Baxter Deutschland GmbH, Unterschleißheim, Germany). Following orotracheal intubation with a 16G cannula (B. Braun Melsungen, Melsungen, Germany), the animals were placed on a heating pad to maintain body temperature, and the intubation tube was connected to a rodent ventilator (SAR 830/P, IITC Life Science Inc., Woodland Hills, CA, USA). The animals were positioned on their right side, and the thorax was shaved. Then, a left lateral thoracotomy in the third intercostal space was performed to gain access to the pulmonary vein. The pulmonary vein was fully ligated or partially ligated to a size of 0.8 or 0.6 mm using silk threads (Seraflex^®^ Silk, 4-0, Serag-Wiessner GmbH & Co. KG, Naila, Germany). Afterward, the chest and the skin incision were closed using standard surgical techniques. Sham animals served as controls and underwent an identical procedure, except for the ligation of the pulmonary vein. To compensate for fluid losses, the rats were given s.c. injections of 1 mL 5% glucose (B. Braun Melsungen, Melsungen, Germany). The schematic diagram of the surgical approach is presented in Figure 10.

### 4.3. Echocardiography

Echocardiographic studies were performed at baseline and one day before the final hemodynamic measurements. Echocardiographic images were acquired using a Vevo 3100 high-resolution imaging system equipped with a 15–30 MHz linear array transducer MX250 (VisualSonics, Toronto, ON, Canada), as described [26]. Briefly, rats were anesthetized using isoflurane (2.5% *v*/*v*, Isofluran Baxter^®^, Baxter Deutschland GmbH, Unterschleißheim, Germany) and placed on a controlled heating table with limbs taped to ECG electrodes. The core temperature was measured via a rectal probe and maintained at 37 °C (Indus Instruments, Houston, TX, USA).

Two-dimensional guided M-mode images were recorded in the parasternal long-axis view. Septal and LV posterior wall thicknesses and LV diameters in end-systole and end-diastole were measured in M-mode from a LV long-axis view at the level of chordae tendinae. The LV ejection fraction (LVEF) was derived using the Teichholz formula. Cardiac output was calculated as the product of the velocity–time integral of the pulsed-wave Doppler tracing of the aortic flow, the aortic cross-sectional area at that level, and the heart rate. Cardiac output was normalized to the body weights of the rats and was presented as cardiac index (CI).

The RV free wall thickness (RVWT) was measured in the right parasternal long-axis view, and the RV internal diameter (RVID) was measured from the apical four-chamber view as the maximal transverse diameter in the middle third of the RV during end-diastole. To assess RV performance, the tricuspid annular plane systolic excursion (TAPSE) was measured in the apical four-chamber view. Images were analyzed offline using the Vevo LAB 5.5.0 software (VisualSonics, Toronto, ON, Canada).

### 4.4. Hemodynamic Measurements

In vivo hemodynamic measurements were performed in separate groups at various time points after PVB (3, 6, 9, and 12 weeks). Rats were anesthetized using isoflurane (3% *v*/*v*) and placed on a controlled heating table, and the core temperature, measured via rectal probe, was maintained at 37 °C. Left ventricular systolic pressure (LVSP) was measured by catheterizing the left ventricle via the right carotid artery. Right jugular vein access was used for RV catheterization to measure RV systolic pressure (RVSP). Hemodynamic measurements were performed using a 2F Mikro-Tip^®^ catheter (SPR-320, Millar Instruments, Houston, TX, USA) and a PowerLab 8/30 System with Chart 7.0 software (AD Instruments GmbH, Spechbach, Germany). Immediately after the completion of the hemodynamic measurements, the rats were exsanguinated under deep isoflurane anesthesia (5% *v*/*v*, Isofluran Baxter^®^, Baxter Deutschland GmbH, Unterschleißheim, Germany), and blood samples were collected.

### 4.5. Lung Fixation, Organ Harvest, and Right Heart Hypertrophy Assessment

Lung fixation and organ harvest were performed as previously described [26]. Briefly, the lungs were flushed with saline (B. Braun Melsungen, Melsungen, Germany) through the pulmonary artery until a white appearance was observed. For molecular biology assessment, the lungs were snap-frozen in liquid nitrogen and stored at −80 °C. For immunohistochemistry, lungs were fixed with formalin (Otto Fischer, Saarbrücken, Germany). The formalin-fixed lungs were subjected to paraffin embedding. For immunofluorescence, lungs were embedded in agarose and fixed in formalin.

Hearts were harvested immediately after the animals were euthanized. The ventricles were dissected free of the great vessels and atria. The RV was separated from the left ventricle (LV) + septum (LV + S). The RV and (LV + S) were patted dry and weighed. The weight ratio RV/(LV + S) was calculated as an index of RV hypertrophy.

### 4.6. Immunohistochemistry

The paraffin-embedded lung tissues were subjected to sectioning to yield 2 µm thick sections. For the assessment of the medial wall thickness (MWT) of pulmonary vessels, elastica-van-Gieson staining was performed, as previously described [27]. The percent medial thickness of muscular vessels (20–50 µm) was calculated using the formula MWT = [(external diameter − internal diameter/external diameter) × 100]. Around 100 pulmonary vessels were analyzed per lung section from each rat. MWT was assessed in the left lungs harvested 3, 6, 9, and 12 weeks after PVB.

The degree of muscularization of peripheral pulmonary vessels was assessed by double staining with an anti-α-smooth muscle actin antibody (dilution 1:700, clone 1A4, Sigma, Saint Louis, MO, USA) and anti-human von Willebrand factor antibody (vWF, dilution 1:1000, Dako, Hamburg, Germany), as described previously [27]. Analysis of the degree of muscularization of the vessels was performed using Qwin 3.0 software (Leica, Wetzlar, Germany). Images were taken using a light microscope CTR6000 (Leica, Wetzlar, Germany). In each rat, 300 small- (25 to 50 µm diameter) and medium-sized vessels (50 to 100 µm diameter) were categorized as muscular, partially muscular, or non-muscular. Pulmonary vessels that contained > 70% of α-actin positive-vessel wall area were defined as fully muscularized; vessels with <4% of α-actin positive-vessel area were defined as non-muscular. Vessels that contained 4–70% of α-actin positive-vessel area were defined as partially muscularized. All analyses were performed in a blinded fashion. The degree of muscularization of peripheral pulmonary vessels was assessed in left lungs harvested 3, 6, 9, and 12 weeks after PVB.

To detect inflammatory cells, sections were deparaffinized and rehydrated. Antigen retrieval was performed by cooking the slides in HIER T-EDTA Buffer pH 9.0 (Zytomed Systems, Berlin, Germany). The slides were washed in TBS buffer (Zytomed Systems GmbH, Berlin, Germany), and unspecific binding was blocked using Rodent Block R (Zytomed Systems GmbH, Berlin, Germany). The slides were incubated with mouse monoclonal primary anti-CD3 (dilution 1:20, Rabbit, Zytomed Systems GmbH, Berlin, Germany) or anti-CD-68 (dilution 1:20, mouse, Bio-Rad, Hercules, CA, USA) antibodies diluted in antibody diluent (Zytomed Systems GmbH, Berlin, Germany) overnight at +4 °C. For visualization, ZytoChem Plus phosphatase polymer kit (Zytomed Systems GmbH, Berlin, Germany) and Warp Red Chromogen substrate kit (Biocare Medical LLC, Concord, CA, USA) were used, following the manufacturer’s protocols. CAT Haematoxylin solution (Biocare Medical LLC) was used for counterstaining. Images were taken using a light microscope CTR6000 (Leica, Wetzlar, Germany).

### 4.7. Prussian Blue Reaction

The Prussian blue reaction for free ionic iron (Fe^3+^) in lung tissue was conducted using the Hematognost Fe^®^ kit (Merck Millipore, Darmstadt, Germany) following the manufacturer’s protocol. Briefly, the slides were incubated in the staining solution (1:1 ratio of 4.78% potassium hexacyanoferrate (II) and 5% hydrochloric acid), rinsed in distilled water and counterstained with nuclear fast red solution. Images were taken using a light microscope CTR6000 (Leica, Wetzlar, Germany).

### 4.8. Tissue Marking Dye

To identify arteries and veins, a green tissue-marking dye (Davidson Marking System, Bradley Products Inc., Minneapolis, MN, USA) was injected through the pulmonary artery, which does not diffuse through the capillary system. Images were taken using a light microscope CTR6000 (Leica, Wetzlar, Germany).

### 4.9. Immunofluorescence

The agarose-embedded lungs were cut into 350 µm thick sections. Endogenous peroxidase activity was quenched through the incubation of sections in 5% hydrogen peroxide in MeOH overnight at room temperature. Unspecific binding was blocked using blocking buffer (BSA in PBS + Triton-X-100 + goat serum) for 2 × 1 h under constant agitation. Afterward, sections were incubated with anti-α-smooth muscle actin antibodies (anti-SMA-Cy3, #C6198, Clone 1A4, Sigma-Aldrich (St. Louis, MO, USA), 1:250 dilution, and anti-SMA-488, #C6198, Clone 1A4, Sigma-Aldrich, 1:250 dilution) and hydrazide-633 (Alexa Fluor 633 Hydrazide bis(triethylammonium) salt, #A30634, Invitrogen (Waltham, MA, USA), 1:500 dilution) in blocking buffer for 3 days at 4 °C under constant shaking. Unbound antibodies were then removed by washing sections five times for 1 h with washing buffer (PBS + Triton-X-100 + goat serum) at room temperature. In the following steps, the sections were post-fixed in 4% formalin for 1 h at 4 °C and washed 2 times with PBS for 20 min. Then, sections were dehydrated, followed by clearing of the tissue with BABB (Benzyl-Alcohol-Benzyl-Benzoate, 1:1, Sigma-Aldrich, #305197, Sigma B6630). Tissue sections were examined using a fluorescence stereomicroscope M205 FCA coupled to DFC9000 GT camera (Leica, Wetzlar, Germany).

### 4.10. Statistical Analysis

All data are presented as mean ± SEM. The statistical analysis was performed using GraphPad Prism (Version 9; GraphPad Software Inc., La Jolla, CA, USA). A two-tailed unpaired Student’s *t* test was used to compare the differences between the groups and a *p*-value of <0.05 was considered statistically significant.

## Data Availability

Data is contained within the article.

## References

[1] Sysol J.R., Machado R.F. (2018). Classification and pathophysiology of pulmonary hypertension. Contin. Cardiol. Educ..

[2] Humbert M., Kovacs G., Hoeper M.M., Badagliacca R., Berger R.M.F., Brida M., Carlsen J., Coats A.J.S., Escribano-Subias P., Ferrari P. (2022). 2022 ESC/ERS Guidelines for the diagnosis and treatment of pulmonary hypertension. Eur. Heart J..

[3] Leung C.C., Moondra V., Catherwood E., Andrus B.W. (2010). Prevalence and risk factors of pulmonary hypertension in patients with elevated pulmonary venous pressure and preserved ejection fraction. Am. J. Cardiol..

[4] Lam C.S., Roger V.L., Rodeheffer R.J., Borlaug B.A., Enders F.T., Redfield M.M. (2009). Pulmonary hypertension in heart failure with preserved ejection fraction: A community-based study. J. Am. Coll. Cardiol..

[5] Adir Y., Guazzi M., Offer A., Temporelli P.L., Cannito A., Ghio S. (2017). Pulmonary hemodynamics in heart failure patients with reduced or preserved ejection fraction and pulmonary hypertension: Similarities and disparities. Am. Heart J..

[6] Bursi F., McNallan S.M., Redfield M.M., Nkomo V.T., Lam C.S., Weston S.A., Jiang R., Roger V.L. (2012). Pulmonary pressures and death in heart failure: A community study. J. Am. Coll. Cardiol..

[7] Valero-Muñoz M., Backman W., Sam F. (2017). Murine Models of Heart Failure with Preserved Ejection Fraction: A “Fishing Expedition”. JACC Basic Transl. Sci..

[8] Liu S.F., Yan Y. (2022). Animal models of pulmonary hypertension due to left heart disease. Anim. Models Exp. Med..

[9] Huston J.H., Shah S.J. (2022). Understanding the Pathobiology of Pulmonary Hypertension Due to Left Heart Disease. Circ. Res..

[10] Arrigo M., Huber L.C. (2013). Eponyms in cardiopulmonary reflexes. Am. J. Cardiol..

[11] Silove E.D., Tavernor W.D., Berry C.L. (1972). Reactive pulmonary arterial hypertension after pulmonary venous constriction in the calf. Cardiovasc. Res..

[12] Wyatt J.P., Burke D.R., Hanlon C.R. (1953). Morphologic Study of Canine Lungs after Ligation of the Pulmonary Veins*. Am. J. Pathol..

[13] LaBourene J.I., Coles J.G., Johnson D.J., Mehra A., Keeley F.W., Rabinovitch M. (1990). Alterations in elastin and collagen related to the mechanism of progressive pulmonary venous obstruction in a piglet model. A hemodynamic, ultrastructural, and biochemical study. Circ. Res..

[14] Aguero J., Ishikawa K., Hadri L., Santos-Gallego C., Fish K., Hammoudi N., Chaanine A., Torquato S., Naim C., Ibanez B. (2014). Characterization of right ventricular remodeling and failure in a chronic pulmonary hypertension model. Am. J. Physiol. Heart Circ. Physiol..

[15] Pereda D., García-Alvarez A., Sánchez-Quintana D., Nuño M., Fernández-Friera L., Fernández-Jiménez R., García-Ruíz J.M., Sandoval E., Aguero J., Castellá M. (2014). Swine model of chronic postcapillary pulmonary hypertension with right ventricular remodeling: Long-term characterization by cardiac catheterization, magnetic resonance, and pathology. J. Cardiovasc. Transl. Res..

[16] Fayyaz A.U., Edwards W.D., Maleszewski J.J., Konik E.A., DuBrock H.M., Borlaug B.A., Frantz R.P., Jenkins S.M., Redfield M.M. (2018). Global Pulmonary Vascular Remodeling in Pulmonary Hypertension Associated with Heart Failure and Preserved or Reduced Ejection Fraction. Circulation.

[17] Garcia-Lunar I., Pereda D., Santiago E., Solanes N., Nuche J., Ascaso M., Bobí J., Sierra F., Dantas A.P., Galán C. (2019). Effect of pulmonary artery denervation in postcapillary pulmonary hypertension: Results of a randomized controlled translational study. Basic Res. Cardiol..

[18] Kato H., Fu Y.Y., Zhu J., Wang L., Aafaqi S., Rahkonen O., Slorach C., Traister A., Leung C.H., Chiasson D. (2014). Pulmonary vein stenosis and the pathophysiology of “upstream” pulmonary veins. J. Thorac. Cardiovasc. Surg..

[19] Cottrill C.M., O’Connor W.N., Fitz R., Gillespie M.N. (1992). Pulmonary vascular remodeling in rats with unilaterally banded lobar pulmonary veins. Cardiol. Young.

[20] Lam C.F., Croatt A.J., Richardson D.M., Nath K.A., Katusic Z.S. (2005). Heart failure increases protein expression and enzymatic activity of heme oxygenase-1 in the lung. Cardiovasc. Res..

[21] Snow J.B., Kanagy N.L., Walker B.R., Resta T.C. (2009). Rat strain differences in pulmonary artery smooth muscle Ca^2+^ entry following chronic hypoxia. Microcirculation.

[22] Stenmark K.R., Meyrick B., Galie N., Mooi W.J., McMurtry I.F. (2009). Animal models of pulmonary arterial hypertension: The hope for etiological discovery and pharmacological cure. Am. J. Physiol. Lung Cell Mol. Physiol..

[23] Karamsetty M.R., Leiter J.C., Ou L.C., Preston I.R., Hill N.S., Yuan J.X.J. (2004). Strain Differences of Hypoxia-Induced Pulmonary Hypertension. Hypoxic Pulmonary Vasoconstriction: Cellular and Molecular Mechanisms.

[24] Ou L.C., Smith R.P. (1983). Probable strain differences of rats in susceptibilities and cardiopulmonary responses to chronic hypoxia. Respir. Physiol..

[25] De Jesus L., Zurita E., Gomez M.d.J., Suarez J. (2015). Different susceptibility to vascular damage induced by chronic hyperglycemia in aortic and pulmonary arteries from Sprague Dawley and Wistar Kyoto rats. FASEB J..

[26] Rai N., Sydykov A., Kojonazarov B., Wilhelm J., Manaud G., Veeroju S., Ruppert C., Perros F., Ghofrani H.A., Weissmann N. (2022). Targeting peptidyl-prolyl isomerase 1 in experimental pulmonary arterial hypertension. Eur. Respir. J..

[27] Amirjanians M., Egemnazarov B., Sydykov A., Kojonazarov B., Brandes R., Luitel H., Pradhan K., Stasch J.P., Redlich G., Weissmann N. (2017). Chronic intratracheal application of the soluble guanylyl cyclase stimulator BAY 41-8543 ameliorates experimental pulmonary hypertension. Oncotarget.

